# Supplementary figures and images for: Epigenetic Suppression of Interferon Lambda Receptor Expression Leads to Enhanced Human Norovirus Replication In Vitro
Source: mBio. 2019 Oct 1;10(5):e02155-19. doi: 10.1128/mBio.02155-19 (PMC6775457; doi:10.1128/mBio.02155-19)

**Fig. S1****A**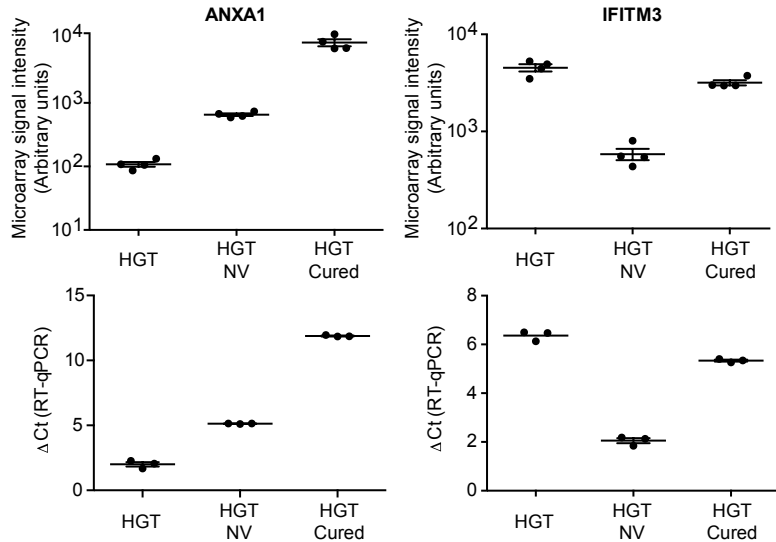**B**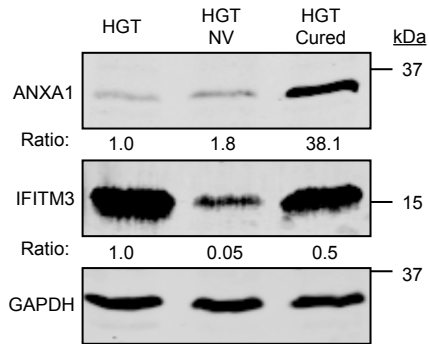

Supplement: FIG S1 [file mBio.02155-19-sf001.pdf]

**Fig. S2****A**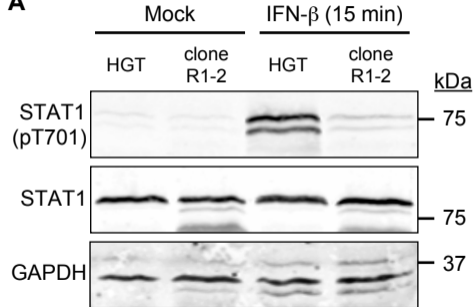**B**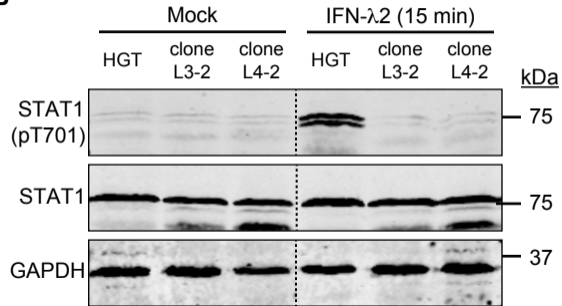

Supplement: FIG S2 [file mBio.02155-19-sf002.pdf]
